# X-search: an open access interface for cross-cohort exploration of the National Sleep Research Resource

**DOI:** 10.1186/s12911-018-0682-y

**Published:** 2018-11-13

**Authors:** Licong Cui, Ningzhou Zeng, Matthew Kim, Remo Mueller, Emily R. Hankosky, Susan Redline, Guo-Qiang Zhang

**Affiliations:** 1 Department of Computer Science, University of Kentucky, Lexington, KY USA; 2 Institute for Biomedical Informatics, University of Kentucky, Lexington, KY USA; 3 Brigham and Women’s Hospital, Boston MA, USA; 4 Harvard Medical School, Harvard University, Boston MA, USA

**Keywords:** Open access, FAIR, Cohort discovery, Hypothesis generation, Data heterogeneity, Data harmonization

## Abstract

**Background:**

The National Sleep Research Resource (NSRR) is a large-scale, openly shared, data repository of de-identified, highly curated clinical sleep data from multiple NIH-funded epidemiological studies. Although many data repositories allow users to browse their content, few support fine-grained, cross-cohort query and exploration at study-subject level. We introduce a cross-cohort query and exploration system, called X-search, to enable researchers to query patient cohort counts across a growing number of completed, NIH-funded studies in NSRR and explore the feasibility or likelihood of reusing the data for research studies.

**Methods:**

X-search has been designed as a general framework with two loosely-coupled components: semantically annotated data repository and cross-cohort exploration engine. The semantically annotated data repository is comprised of a canonical data dictionary, data sources with a data dictionary, and mappings between each individual data dictionary and the canonical data dictionary. The cross-cohort exploration engine consists of five modules: query builder, graphical exploration, case-control exploration, query translation, and query execution. The canonical data dictionary serves as the unified metadata to drive the visual exploration interfaces and facilitate query translation through the mappings.

**Results:**

X-search is publicly available at https://www.x-search.net/with nine NSRR datasets consisting of over 26,000 unique subjects. The canonical data dictionary contains over 900 common data elements across the datasets. X-search has received over 1800 cross-cohort queries by users from 16 countries.

**Conclusions:**

X-search provides a powerful cross-cohort exploration interface for querying and exploring heterogeneous datasets in the NSRR data repository, so as to enable researchers to evaluate the feasibility of potential research studies and generate potential hypotheses using the NSRR data.

## Background

Sharing and reusing biomedical digital data have gained increasing attention to accelerate scientific discovery and enhance research reproducibility [1–4]. Various data repositories have been developed and made available for biomedical researchers, such as UniProt – a comprehensive resource for protein sequence and annotation data [5], GDC – the National Cancer Institute’s Genomic Data Commons [6], BioPortal – a repository of biomedical ontologies [7], OpenfMRI – a repository for sharing task-based fMRI data [8], and NSRR – the National Sleep Research Resource [9–11].

A widely endorsed set of guiding principles for biomedical data management in data repositories is FAIR: making data Findable, Accessible, Interoperable, and Reusable [4]. The FAIR principles are essential for researchers to find the data of interest, which may be further reused for generating or testing hypotheses. While most existing data repositories enable researchers to browse and download data – sometimes under data use or regulatory constraints, very few allow users to freely and openly perform fine-grained, cross-dataset query and exploration of study-subject level before deciding which specific datasets to gain further access. Such fine-grained data exploration capabilities allow users to compose complex queries over a large number of cohorts, quickly assess the feasibility of research studies or generate/test potential hypotheses, and then make appropriate full data access requests.

In this paper, we introduce a deployed, web-based cross-cohort query and exploration tool, called X-search, enabling sleep researchers to query patient cohort counts across datasets in the NSRR data repository [11] and explore the feasibility or likelihood of sleep-related research studies. NSRR is one of the largest data repositories for sharing de-identified sleep research data (including clinical data elements and physiological signals), collected from multiple NIH-funded epidemiological cohort studies and clinical trials. NSRR contains semantically annotated data of over 26,000 human subjects. The NSRR data repository was implemented following the FAIR principles. The X-search engine has been developed to make the clinical data elements in NSRR more findable – in the sense that researchers can perform cross-cohort queries and exploration over heterogeneous datasets to evaluate the feasibility of potential studies.

There have been systems developed for querying patient cohorts across multiple data sources, such as the Shared Health Research Information Network (SHRINE) [12] through i2b2 federation [13], VISual AGgregator and Explorer (VISAGE) [14], and an adaptable architecture defined by Bache et al. [15]. However, these systems were developed for access by approved and registered users, and none of them are available to explore publicly available data sharing repositories like NSRR. X-search not only supports querying of subject counts, but also provides more data exploration functionalities (including graphical exploration and case-control exploration) to make data more findable, accessible, and through NSRR, more interoperable and reusable.

## Methods

**Fig. 1:**
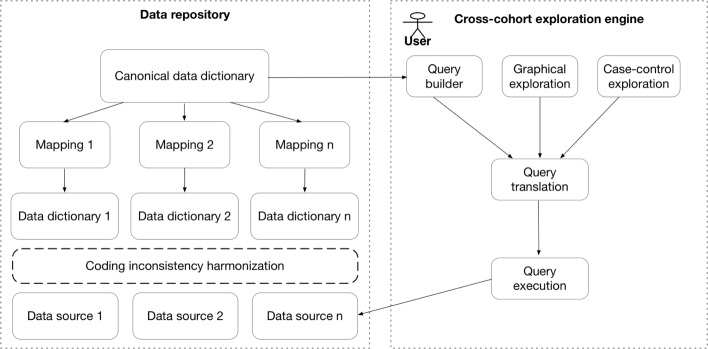
High-level architecture. Left: Open data repository with heterogeneous data sources. Right: The cross-cohort exploration system

Our overall objective is to create an open-access query interface that allows a user to perform aggregated search on NSRR content before requesting full access to specific datasets. To do so, we designed the system architecture to be comprised of two major components: a semantically annotated data repository with heterogeneous datasets (Fig. 1, left) and the cross-cohort exploration engine (Fig. 1, right).

We assume that the datasets (or data sources) are in the structured format which can be loaded to relational databases such as MySQL. Each data source has a dedicated data dictionary originated from the source study and available for open access through NSRR. The data dictionary describes the data elements in the dataset, including names, definitions, data types, units, and value domains (or allowable values). The canonical data dictionary consists of core query terms (or common data elements) across different datasets. Here a common data element is a data element that is common to multiple NSRR data sets. Each mapping involves the data element mappings between the source data dictionary and the canonical data dictionary.

The cross-cohort exploration engine consists of five components: query builder, graphical exploration, case-control exploration, query translation, and query execution. The query builder, graphical exploration, and case-control exploration components involve visual interfaces and use the canonical data dictionary as the query and exploration terms. The query translation module translates a user query composed in the query builder to the actual query statement in SQL. The query execution module connects to each database (or data source) and executes the query statement.

### Data repository

#### Data sources and data dictionaries

We used nine datasets in the NSRR data repository: Sleep Heart Health Study (SHHS), Childhood Adenotonsillectomy Trial (CHAT), Heart Biomarker Evaluation in Apnea Treatment (HeartBEAT), Cleveland Family Study (CFS), Study of Osteoporotic Fractures (SOF), Osteoporotic Fractures in Men Study (MrOS), Cleveland Children’s Sleep and Health Study (CCSHS), Hispanic Community Health Study/Study of Latinos (HCHS/SOL), and Multi-Ethnic Study of Atherosclerosis (MESA). Each dataset may involve multiple patient visits, where the actual data records for each visit is stored in a comma-separated values (CSV) file.

Each data source has a data dictionary semantically defining the scope and characteristics of data elements (or variables) in the dataset, including the short name, display name, description, type, unit, domain, and synonyms. The short name of a variable is the actual column name used in the database storing the dataset, and serves as the unique identifier for a variable (note that one dataset may have the same variable in different patient visits). The display name is the variable name shown to users in the visual interface, and it is more informative than the short name. The description provides more detailed information about the variable such as meta information. The type defines the data type of a variable, such as identifier, numeric, and categorical. The unit is applicable to numeric variables describing a measurable quantity. The domain specifies the allowable values for a categorical variable. The label stores the synonyms or indexed terms which can be used to retrieve the variable. The data dictionary containing the above-mentioned information can be structurally specified in CSV files.

#### Canonical data dictionary and mappings

The canonical data dictionary specifies the common data elements across different data sources. These common data elements serve as the core query terms in the visual interface for users to browse or search. The core terms capture demographic information (e.g., age, gender, race), anthropometric parameters (e.g., body mass index, height, weight), physiological measurements (e.g., heart rate, diastolic blood pressure, systolic blood pressure), medical history (e.g., asthma, atrial fibrillation, insomnia, sleep apnea), medications (e.g., benzodiazepine, estrogen, progestin), sleep study data (e.g., sleep duration, quality of sleep, obstructive sleep apnea events, apnea hypopnea index, average oxygen saturation during sleep), and laboratory data (e.g., HDL cholesterol, creatinine). In addition, these data elements are mapped and linked to the NIH Common Data Element (CDE) repository [16] if applicable.

Since a data element in the canonical data dictionary may have different variable names in disparate data sources, there is a need for a mapping from each individual data source to the canonical data dictionary. Therefore, for each data source, a uniform mapping template is utilized to map the source data elements to the common data elements (*m* to 1 mapping). Take the common data element “body mass index" as an example, there are two data elements in the SHHS dataset mapping to it (“bmi_s1" in the baseline visit, and “bmi_s2" in the followup visit); and there are two data elements in the HeartBEAT dataset mapping to it (“bmi_scrn" and “calc_bmi" in the baseline visit).

#### Coding inconsistency harmonization

A unique challenge in exploring data in multiple heterogeneous data sources is to address the coding inconsistency issue, which involves the detection and harmonization of inconsistencies among the disparate value domains for the same data element. Such inconsistencies occur frequently for categorical variables. For instance, the “gender" variable has inconsistent codings across the nine datasets in NSRR (see Table 1), where 1 represents “Male" and 2 represents “Female” in SHHS, whereas 1 represents “Male” and 0 represents “Female” in HeartBEAT, and 1 represents “Female” and 2 represents “Male” in MrOS. Such heterogeneity must be harmonized to ensure that data exploration activities obtain accurate results. In order to achieve this, an automated program has been developed to detect the inconsistent codings among different datasets by leveraging the canonical data dictionary, each source data dictionary, and the mappings between source data dictionaries and the canonical data dictionary.

**Table 1:** Harmonizing coding inconsistencies among different datasets for the “gender” variable

Dataset	Code	Name	Harmonized
SHHS	1	Male	1 - Male
	2	Female	2 - Female
CHAT	1	Male	1 - Male
	2	Female	2 - Female
HeartBEAT	0	Female	2 - Female
	1	Male	1 - Male
CFS	0	Female	2 - Female
	1	Male	1 - Male
MrOS	1	Female	2 - Female
	2	Male	1 - Male
CCSHS	0	Female	2 - Female
	1	Male	1 - Male
HCHS	0	Female	2 - Female
	1	Male	1 - Male
MESA	0	Female	2 - Female
	1	Male	1 - Male

To harmonize the detected inconsistencies, manual review has been involved to define the uniform codings and create appropriate mappings from heterogeneous codings to the uniform coding. Take the “gender” codings in Table 1 as an example, a uniform coding with 1 representing “Male” and 2 representing “Female” can be defined, and heterogeneous codings in the original data sources can be mapped to the uniform coding (each row is a such mapping). This harmonization step is essential to ensure the data interoperability across disparate data sources.

#### Data loading

To support the cross-cohort exploration activities, we need to import the actual data in each dataset, load common data elements in the canonical data dictionary and mappings, and harmonize coding inconsistencies. This process is described in the following three steps.

In Step 1, for each dataset, we create a relational database in MySQL to store the actual data; and we leverage the data dictionary of the dataset to automatically create tables in the database and load actual data into tables. More specifically, the data type of a data element specified in the data dictionary (e.g., decimal) seamlessly determines the data type of a column in a MySQL table (e.g., DOUBLE), which enables automated creation of MySQL statements to create tables and insert data records in CSV files to the tables.

In Step 2, we import common data elements in the canonical data dictionary to the backend database of the X-search web application, as well as the mappings between the data elements in each dataset and the common data elements. Such information will be leveraged to support the query translation of the web-based query widgets to the backend MySQL query statements.

In Step 3, we handle coding inconsistencies for heterogeneous data elements that are mapped to the same common data element. There are two options: (1) harmonize the actual data in each dataset according to the uniform codings, so that the query translation step can directly use the uniform codings to create common SQL query statements for disparate datasets; (2) keep the actual data in each dataset as is, and rely on the query translation step to utilize the mappings to the uniform codings to generate different SQL query statements for disparate datasets. Although the latter option saves time and effort to update the loaded data, the former option saves the query time due to less hassle on the query translation step. In this paper, we have adopted the first option to speed up the query translation process and reduce users’ waiting time when performing data exploration activities.

### The X-search cross-cohort exploration engine

#### Query builder

A powerful and intuitive interface, called query builder, has been designed and developed to enable researchers to quickly find the right common data elements and perform exploratory cross-cohort query. The query builder consists of the four areas which are corresponding to four steps to perform cross-cohort queries as follows. (1) Area to select datasets, where users can choose a collection of datasets to focus on; (2) Area to add query terms, where users can look for query terms (or common data elements) of interest; (3) Area to construct queries, where query criteria can be specified for each query term; (4) Area for query results, where retrieved subject counts satisfying query criteria are returned to users.

In the area to add query terms, there are two modes to look for common data elements of interest: browsing and search. The browsing mode provides a hierarchical view of query terms so that novice users can explore all the available common data elements level by level. The search mode enables expert users to directly search for query terms of interest. The query builder uses a dynamic mechanism to generate visual query widgets corresponding to selected query terms. For instance, selecting a categorical term generates a widget with checkboxes for specifying possible values; and selecting a numerical term generates a widget with a slider bar for specifying a range of values.

The query builder interface is a general design such that each area serves as a placeholder where the content of each area can be filled automatically with research data in different domains. In this work, the area to select datasets is filled with the names of the nine datasets from NSRR, the area to add query terms consists of the canonical data dictionary terms, the area to construct queries contains dynamic query widgets corresponding to the selected query terms, and the area for query results is driven by the query criteria specified in the area to construct queries.

#### Graphical exploration

The graphical exploration interface has been designed to support visual exploration of two common data elements (say *x* and *y* corresponding to *x*-axis and *y*-axis). The graphical views include bar plots and box plots. Bar plots are shown when the *y*-axis is a categorical common data element, and box plots are displayed when the *y*-axis is a numeric common data element. Such plots enable users to have a better understanding of the data distribution of *y* against *x*. Since there may be multiple variables in each individual dataset that are mapped to *x* or *y*, multiple plots are generated for each pair of variables.

#### Case-control exploration

The case-control exploration interface allows registered users to perform cross-cohort case-control analyses. It provides a general template for users to build a case-control exploration step by step. Step 1 is to set base query terms, where users can specify the criteria for base population (e.g., age between 45 and 85 years, body mass index between 30 and 85 kilograms per square meter, and no history of cardiovascular disease). Step 2 is to set the condition for cases (e.g., had diabetes). Step 3 is to set the condition for controls (e.g. no history of diabetes). Step 4 is to set the match terms (e.g., gender and ethnicity). Step 5 is to set outcome terms (e.g., obstructive sleep apnea). The result of the case-control exploration is displayed as a table with case and control counts for the match and outcome terms.

#### Query translation and execution

The query translation module automatically translates the user’s specifications captured by the visual interface into actual MySQL statements to query backend databases. The translation relies on the query terms and values specified in the visual interface, as well as the mappings between each individual data dictionary and the canonical data dictionary. The query statements for multiple data sources are distinct, since these data sources have different table and column information mapping to a common data element.

For each type of common data elements, a general template is predefined and used for dynamically generating the actual MySQL statement for query translation. For example, the general template for querying a numeric common data element with a specified range (min, max) is predefined as:

SELECT¡COUNT¡(DISTINCT¡<mapping.table. identifier>) FROM¡<mapping.table> WHERE¡<mapping.column>¡BETWEEN¡<min>¡AND¡<max>; 

Here <mapping.table> and <mapping.column> represent the data source table and column to which the common data element is mapped to in a dataset. All the variables in the angle brackets can be replaced by real values to generate the actual MySQL statements for different datasets.

The translated MySQL statements are sent to the corresponding data sources to perform the query execution. For the query builder interface, the query execution returns numeric counts of potentially eligible subjects satisfying the query criteria. For the graphical exploration interface, the query execution returns the actual values of data elements for eligible subjects, which are further plotted visually. For the case-control interface, the query execution returns the actual values of data elements for both cases and controls, which are further processed to generate the table-format view with case- and control-counts displayed for the match and outcome terms.

## Results

### Data repository

We used MySQL databases to store the nine datasets. Table 2 lists the names of the datasets, the names of the visits, the numbers of data elements (or variables), the numbers of subjects, and the numbers of mapped variables to the canonical data dictionary. Note that the mapped variables in each visit of a dataset is a subset of all the variables in the visit. The canonical data dictionary contained a total of 919 common data elements (554 of them are specific to the sleep research domain and 365 of them are common across study domains). Among them, 42 were detected to have inconsistent codings across different datasets, including “gender,” “race,” “history of asthma,” and “history of sleep apnea.” A total of 830 mappings from heterogeneous codings to the uniform codings were created to harmonize the data with inconsistent codings. In addition, 57 elements in the canonical data dictionary were linked to the NIH CDE.

**Table 2:** Summary information for each of the nine datasets

Dataset	Visit(s)	No. of variables	No. of subjects	No. of mapped variables
SHHS	shhs1	1266	5804	615
	shhs2	1302	4080	592
CHAT	baseline	2897	464	826
	followup	2897	453	823
HeartBEAT	baseline	859	318	158
	followup	731	301	103
CFS	visit5	2871	735	1023
SOF	visit8	1114	461	350
MrOS	visit1	479	2911	261
	visit2	507	2911	222
CCSHS	trec	143	517	94
HCHS	sol	404	16,415	97
	sueno	505	2252	5
MESA	sleep	723	2237	512

### Cross-cohort exploration engine

We implemented the X-search cross-cohort exploration engine using Ruby on Rails, an agile web development framework. It has been deployed at https://www.x-search.net/ and open to public access for free [17].

**Fig. 2:**
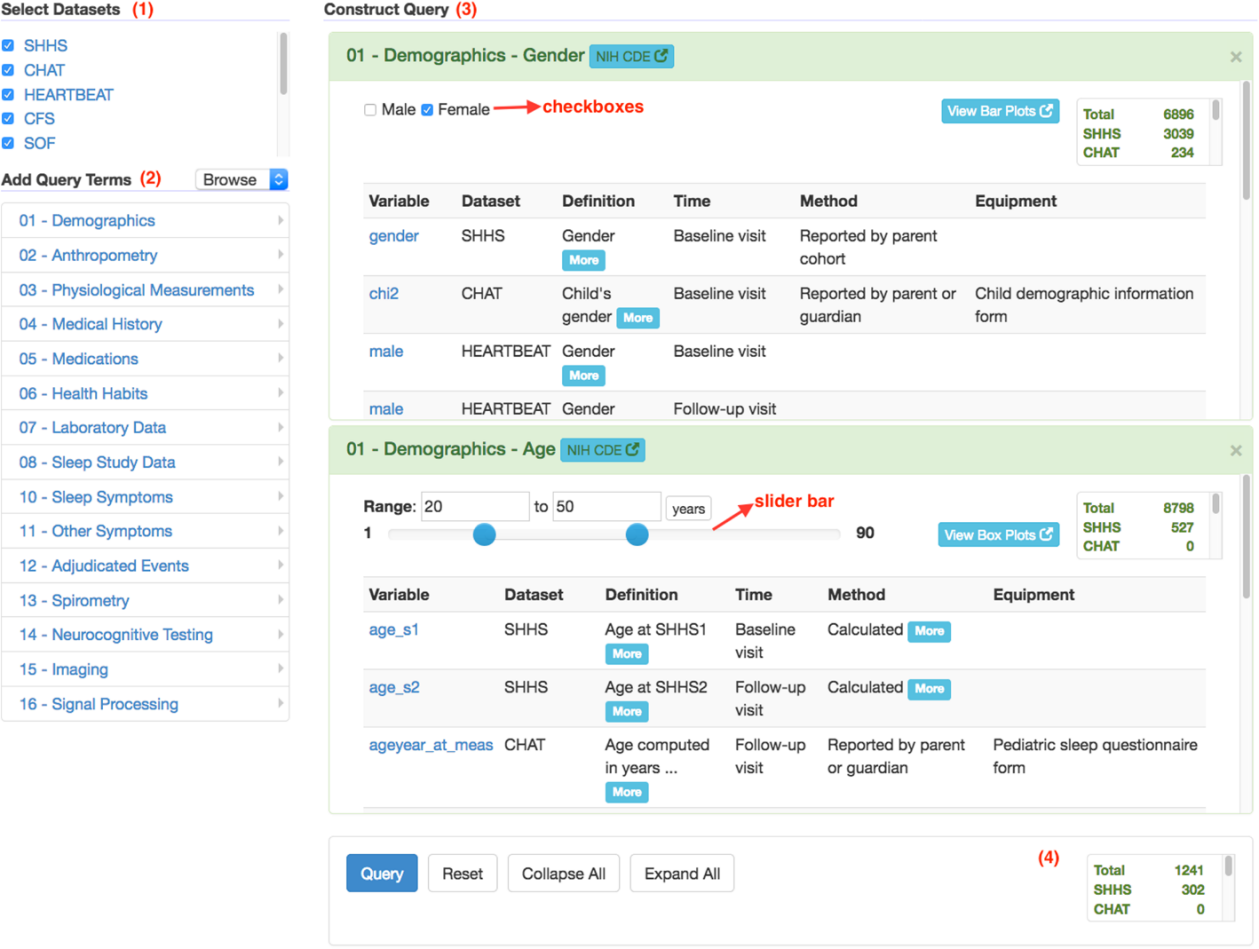
Screenshot of the query builder interface. Four areas: (1) Select Datasets; (2) Add Query Terms; (3) Construct Query; (4) Query Results. This example queries the numbers of female patient subjects aged between 20 and 50

Figure 2 shows the query builder interface with the four areas annotated. In the area to select datasets, all the nine datasets are chosen – five of them can be directly seen, and other four can be seen when scrolling down. The area to construct queries contains two query widgets for “gender” (with checkboxes) and “age” (with a slider bar), with specified query criteria: female, and age between 20 and 50. The area for query results shows the numbers of subject counts meeting the query criteria in each dataset, as well as the total number of subject counts.

**Fig. 3:**
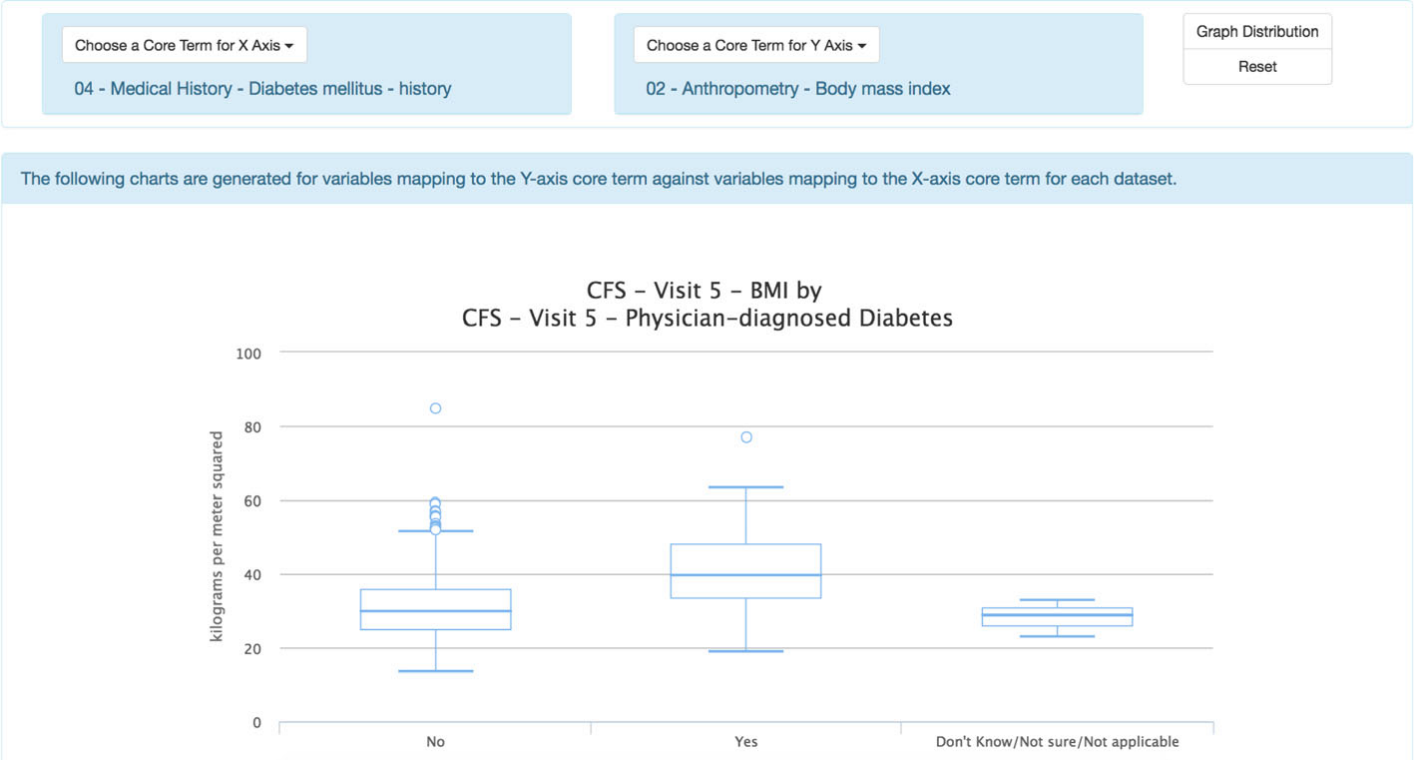
Screenshot of the graphical exploration interface. This example shows one of the box plots generated for body mass index (BMI) against diabetes

Figure 3 gives an example of the graphical exploration interface, where the term for the *y*-axis is specified as “body mass index" and the term for the *x*-axis is “history of diabetes". The box plot shown in the figure is generated based on two variables in the CFS dataset mapped to “body mass index" and “history of diabetes" respectively, and indicates that the median body mass index of patients who had history of diabetes is greater than that of patients who had no history of diabetes.

**Fig. 4:**
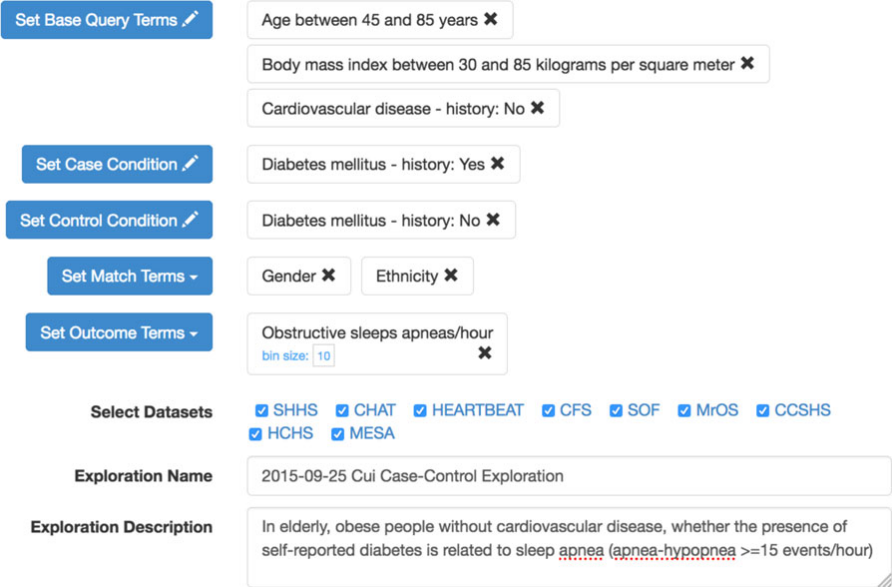
Screenshot of the case-control exploration interface. This example is to explore: In elderly, obese people without cardiovascular disease, whether the presence of self-reported diabetes is related to sleep apnea (apnea-hypopnea >=15 events/hour)

Figure 4 shows the case-control exploration interface illustrating the exemplar steps mentioned in the Methods section. This example is to explore: In elderly (*base query: age between 45 and 85 years*), obese people (*base query: body mass index between 30 and 85*) without cardiovascular disease (*base query: no history of cardiovascular disease*), whether the presence of self-reported diabetes (*case condition: had history of diabetes, control condition: no history of diabetes*) is related to sleep apnea (*outcome term: obstructive sleep apneas/hours*).

The cross-cohort exploration system supports additional functionalities, including the query manager, case-control manager, and International Classification of Sleep Disorders (ICSD) query builder. Query and case-control managers allow users to save queries and case-control explorations for reuse. ICSD query builder is a dedicated query builder for more complicated ICSD terms.

### Usage

Dated to September 4th, 2017, the cross-cohort exploration system has received 1,835 queries from users in a wide range of geographical regions (16 countries), including Australia, Canada, China, France, India, South Africa, United Kingdom, and the United States.

**Fig. 5:**
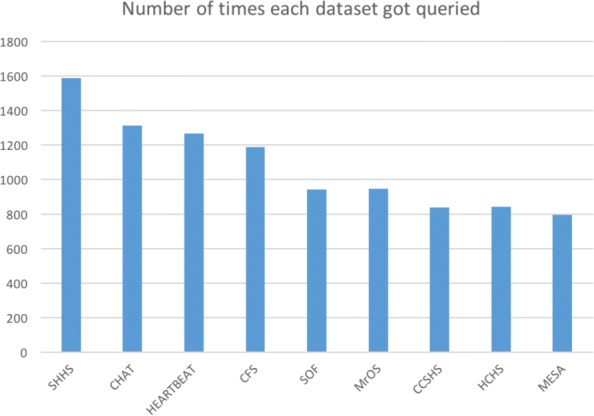
Numbers of times each dataset got queried

Figure 5 shows the number of times each of the nine datasets got queried (note that each user query may involve multiple datasets). And the top ten query terms are: “age," “obstructive sleep apneas/hour,” “central sleep apneas/hour,” “gender," “body mass index," “diabetes mellitus - history," “cardiovascular disease - history," “apnea hypopnea index greater than or equal to 15," “apnea hypopnea index," and “race."

### Use case of exploratory analysis and discovery

To illustrate how the cross-cohort exploration system can be used for exploratory analysis and discovery, we present a use case of using the query builder to determine the comorbidity of *obstructive sleep apnea* with four medical history conditions. We chose two conditions (*hypertension* and *diabetes*) that have been classically associated with obstructive sleep apnea [18, 19] and two conditions (*depression* and *anxiety*) for which the relationship has been less well characterized [20]. Based on clinical diagnosis guidelines [21], obstructive sleep apnea was indicated if a subject had ≥ 5 obstructive sleep apnea events per hour recorded during any of their visits.

Using query builder, we identified 2774 patients from four datasets (SHHS, CHAT, HeartBEAT, and CFS) that met this criterion for obstructive sleep apnea and had information regarding the medical history conditions of interest. Table 3 lists demographic characteristics, including gender and race of the patients with obstructive sleep apnea in each of the datasets (except race in HeartBEAT which is not available yet).

**Table 3:** Demographic characteristics of patients that met criterion (≥ 5 obstructive sleep apnea events/hour) for obstructive sleep apnea (OSA) listed by dataset

	SHHS	CHAT	HeartBEAT	CFS	Total
Obstructive sleep apnea (OSA)	2071	214	300	189	2774
Male	1117 (53.9%)	96 (44.9%)	221 (73.7%)	107 (56.6%)	1541 (55.6%)
Female	954 (46.1%)	118 (55.1%)	79 (26.3%)	82 (43.4%)	1,233 (44.4%)
White	1838 (88.7%)	55 (25.7%)	–	70 (37.0%)	1963 (79.3%)
Black	141 (6.8%)	139 (65.0%)	–	118 (62.4%)	398 (16.1%)
Other	92 (4.4%)	20 (9.3%)	–	1 (0.5%)	113 (4.6%)

Table 4 shows the prevalence of obstructive sleep apnea among patients in the datasets with a history of the medical condition of interest. As seen in Table 4, obstructive sleep apnea was highly prevalent in patients with a history of hypertension and diabetes, consistent with prior reports of the comorbidity between these diseases [18, 19]. We also found that about 60% of patients with a history of depression and anxiety had obstructive sleep apnea, consistent with the higher range of estimates reported previously [20]. Notably, each of the prevalence estimates are on the high end of published findings likely due to the dataset samples having been recruited to investigate obstructive sleep apnea. These samples could be further used to delineate relationships among specific risk or protective factors, demographics, interventions (e.g. medications), and health habits underlying these comorbidities.

**Table 4:** Total number of patients, listed by dataset, that met criterion for obstructive sleep apnea (OSA)

	SHHS	CHAT	HeartBEAT	CFS	Total	Prevalence
Obstructive sleep apnea (OSA)	2071	214	300	189	2774	
Hypertension	2441	2	298	285	3026	
Hypertension and OSA	1330	1	281	105	1717	56.7%
Diabetes	405	0	153	153	711	
Diabetes and OSA	153	0	144	48	345	45.8%
Depression	–	6	100	120	226	
Depression and OSA	–	3	94	38	135	59.7%
Anxiety	–	9	64	60	133	
Anxiety and OSA	–	5	59	16	80	60.2%

Within each dataset, the number of patients with a history of hypertension, diabetes, depression, and anxiety are listed followed by the number of patients with OSA and the condition of interest. On the right is the prevalence of patients with obstructive sleep apnea and a history of the medical condition of interest

## Discussion

### Generalization

Although our X-search was developed for the NSRR, its framework (Fig. 1) has been designed and implemented to be generally applicable to other domains for exploring research data from multiple heterogeneous data sources. For a new domain, the cross-cohort exploration system can be readily used once the data sources, canonical data dictionary and mappings are provided according to the need of the new domain.

### Comparison to related work

To support fast generation of hypotheses and assessment of the feasibility of research studies, various cohort discovery tools have been developed to facilitate the identification of potential research subjects satisfying certain characteristics.

Murphy et al. [13] have developed a cohort selection (or counting) system for the Informatics for Integrating Biology and the Bedside (i2b2) project, which has been widely adopted for querying the count of eligible patients in a single clinical data repository. To support patient cohort identification from multiple data sources, Weber et al. [12] have developed the Shared Health Research Information Network (SHRINE) based on i2b2. SHRINE requires the underlying data sources to have the same data structure based on i2b2. Distinct from SHRINE, our X-search was designed to query multiple data sources with heterogeneous data structures.

Bache et al. [15] defined and validated an adaptable architecture (we refer to it as Bache’s architecture) for identifying patient cohorts from multiple heterogeneous data sources. Bache’s architecture supports multiple data sources with heterogeneous data structures, and handles the heterogeneity in the query translation step. Our X-search differs in that it handles the data heterogeneity in the data loading step, which saves users’ waiting time for query translation.

Zhang et al. [14] have designed and implemented a query interface VISAGE (VISual AGgregator and Explorer) for query patient cohorts. Our X-search shares a similar visual interface design with VISAGE (e.g., checkboxes for categorical variables, and slider bar for numerical variables), but differs from VISAGE in that it adapts a data warehouse approach to harmonize data sources before querying rather than a federated approach to directly query the data sources.

In addition, the above-mentioned systems have been designed for private use in clinical settings, while X-search has been designed for open public use for free in the setting of making data elements more findable in data sharing repositories. To the best of our knowledge, X-search is the first cross-cohort exploration system that is open to public access. Also, more data exploration functionalities (including graphical exploration and case-control exploration) have been provided in X-search.

Another related work is the Observational Medical Outcomes Partnership (OMOP) Common Data Model (CDM) [22] for representing healthcare data from diverse sources in a standardized way, which is open-source and maintained by an international collaborative, Observational Health Data Sciences and Informatics (OHDSI) program [23]. OMOP CDM standardizes data structure and common vocabularies (e.g., SNOMED CT, ICD9CM, RxNorm) across disparate sources, such as electronic health records, administrative claims, and clinical data. A natural question would be whether the OMOP CDM could be directly used for modeling NSRR datasets. However, significant effort needs to be made to transform NSRR datasets into utilization of standardized vocabularies, and there may not be direct transformation due to the fine-grained, sleep-related data elements. It would be interesting to explore the generalizability of OMOP CDM using the NSRR datasets.

There are existing tools on standardizing and harmonizing data elements for clinical research studies such as eleMAP [24] and D2Refine [25], which enable researchers to harmonize local data elements to existing metadata and terminology standards such as the caDSR (Cancer Data Standards Registry and Repository) [26] and NCI Thesaurus [27]. Such tools may be useful for us to map NSRR data elements to existing standards.

### Data access

It is worth noting that X-search only returns subject counts or charts of the query results. It provides an open access interface for users to explore and find data of interest without the need of downloading data in advance. Once users find data of interest, they can gain access to the actual data for further analyses and reuse after completing the NSRR Data Access and Use Agreement online and getting approved.

### Limitations and future work

X-search uses MySQL databases to load and store the actual datasets. However, a limitation of the MySQL database is the restriction on the maximum number of columns in a table. For clinical data with a large number of data elements (e.g., SHHS), split is needed to store all the data which may cause overhead on querying across multiple tables. It would be interesting to use NoSQL databases to store and query NSRR datasets, and compare the performance of the NoSQL- and SQL-based approaches. In addition, we plan to explore how to expand our X-search cross-cohort exploration tool to support the OMOP Common Data Model.

## Conclusions

In this paper, we presented X-search, NSRR’s cross-cohort exploration system to query patient cohort counts across heterogeneous datasets in the National Sleep Research Resource. X-search has received queries from 16 countries, and enabled researchers to perform cross-cohort query and exploration to evaluate the feasibility of potential research studies using shared data in the NSRR repository.

## Abbreviations

CCSHS: Cleveland children’s sleep and health study
CFS: Cleveland family study
CHAT: Childhood Adenotonsillectomy trial
CSV: Comma-separated values
FAIR: Findable, accessible, interoperable, and reusable
HCHS/SOL: Hispanic community health study/study of Latinos
HeartBEAT: Heart Biomarker evaluation in Apnea treatment
MESA: Multi-ethnic study of Atherosclerosis
MrOS: Osteoporotic fractures in men study
NSRR: National sleep research resource
SHHS: Sleep heart health study
SHRINE: Shared health research information network
SOF: Study of osteoporotic fractures
X-search: NSRR cross dataset query interface
